# Exploring Machine Learning Techniques to Predict the Response to Omalizumab in Chronic Spontaneous Urticaria

**DOI:** 10.3390/diagnostics11112150

**Published:** 2021-11-20

**Authors:** Davide Stefano Sardina, Giuseppe Valenti, Francesco Papia, Carina Gabriela Uasuf

**Affiliations:** 1 Allergy Disease Center “Prof. Giovanni Bonsignore”, Institute for Research and Innovation Biomedicine (IRIB), Italian National Research Council (CNR), 90145 Palermo, Italy; davidestefano.sardina@gmail.com (D.S.S.); dott.francesco.papia@gmail.com (F.P.); 2 Allergology and Pulmonology Unit, Provincial Outpatient Center of Palermo, 90129 Palermo, Italy; valentiasma@tiscali.it

**Keywords:** chronic spontaneous urticaria, omalizumab, machine learning technique, biomarkers, anti-IgE

## Abstract

Background: Omalizumab is the best treatment for patients with chronic spontaneous urticaria (CSU). Machine learning (ML) approaches can be used to predict response to therapy and the effectiveness of a treatment. No studies are available on the use of ML techniques to predict the response to Omalizumab in CSU. Methods: Data from 132 CSU outpatients were analyzed. Urticaria Activity Score over 7 days (UAS7) and treatment efficacy were assessed. Clinical and demographic characteristics were used for training and validating ML models to predict the response to treatment. Two methodologies were used to label the data based on the response to treatment (UAS7 ≥ 6): (A) at 1, 3 and 5 months; (B) classifying the patients as early responders (ER), late responders (LR) or non-responders (NR) (ER: UAS 7 ≥ 6 at first month, LR: UAS 7 ≥ 6 at third month, NR: if none of the previous conditions occurred). Results: ER were predominantly characterized by hypertension, while LR mainly suffered from asthma and hypothyroidism. A slight positive correlation (R^2^ = 0.21) was found between total IgE levels and UAS7 at 1 month. Variable Importance Analysis (VIA) reported D-dimer and C-reactive proteins as the key blood tests for the performance of learning techniques. Using methodology (A), SVM (specificity of 0.81) and k-NN (sensitivity of 0.8) are the best models to predict LR at the third month. Conclusion: k-NN plus the SVM model could be used to identify the response to treatment. D-dimer and C-reactive proteins have greater predictive power in training ML models.

## 1. Introduction

Chronic spontaneous urticaria (CSU) is defined by the spontaneous occurrence of wheals, angioedema or both that last longer than 6 weeks. Globally, it affects 1% of the general population, has an unpredictable course and duration, and 11–14% of the patients suffer for more than 5 years [1]. Moreover, the impaired quality of life of these patients has a dramatic impact on daily life, personal relationships, work and sleep [2,3].

Appropriate effective treatment is, therefore, extremely important. According to the EAACI/GA^2^LEN/EDF/WAO guideline for urticaria, the first-line therapy is second-generation H1-antihistamines in standard dose, but, unfortunately, these are effective in less than 50% of CSU patients [4]. The third-line therapy, omalizumab, an anti-IgE monoclonal antibody, is more effective with a complete response rate that ranges from 26% to 83%, as demonstrated in several landmark studies [5,6,7,8,9].

Ideally, the treatment of patients with CSU should be “tailored to patient’s” clinical or biochemical characteristics, based on predictors of response to treatment.

Identification of these predictors will save time, costs and improve patient’s lifestyle.

Machine Learning (ML) approaches are bioinformatic techniques that use labeled data to try to identify significant patterns called “supervised” with the purpose to create a statistical model to explain an unresolved question. Once the model is created, these algorithms are able to predict the class for new data whose label is unknown. Each label is associated with a set of features, which usually are able to explain the model. In this study, CSU patients are labeled as early, late and non-responders while the features used to train the model were several characteristics, such as demographic and clinical parameters etc., associated to each patient.

It is well known that algorithms have been used in medicine [10], to detect anaphylaxis cases [11], in human microbiome studies [12], in anesthesiology [13], obesity [14] and drug discovery [15], and other medical fields [16]. At the moment, nobody has assessed the performances of well-known ML approaches for the prediction of treatment response with omalizumab in patients with CSU.

## 2. Methods

### 2.1. Participants

From October 2018 to December 2019, database was retrospectively collected from 132 South Italian CSU outpatients recruited from the Allergy Disease Center “Prof. Giovanni Bonsignore” and the Allergology and Pulmonology Unit of Palermo. Urticaria activity was assessed using the UAS7. As response threshold, UAS7 was considered equal or greater than 6 to classify CSU patients as “early responders” (ER; if they started to respond at 1 month and remained in this condition until the fifth month), and “late responders” (LR; if the response was achieved at the third month). If none of the previous conditions occurred, the patients were considered as “non-responders” (NR). The classification of our cohort is described in Table 1.

Several baseline variables were collected, e.g., age, sex, residence, weight, height, urticaria start date, co-occurrence of angioedema, total serum IgE (UI/mL), total number (k/uL) and mean basophils number/mm^3^ [17], D-dimer (ng/mL), C-reactive protein (mg/L), co-occurrence of allergies or other concomitant diseases, pre-treatment outlier blood test exams, basophil activation test (BAT) results, UAS and UAS7 pre-treatment, after 1 month, 3 and 5 months.

### 2.2. Machine Learning Approach

Support Vector Machine (SVM) [18] is a widely used classification algorithm. SVM learns from data trying to find the best hyperplane in a high-dimensional space that is capable of dividing them in different classes. In this direction, kernel function provides a methodology to deal with non-linearly separable data. The algorithm is based on support vectors representing actual data maximizing the distance between each class from the hyperplane.

k-nearest neighbors (k-NN) considers the characteristics of closest objects to classify new input data. New cases are labeled according to a voting technique by using the most common class among its k neighbors [19]. Generally, k = 1 is a good choice indicating that only 1 neighbor is considered, although with larger dataset higher values of k can be used.

Cross validation method is used to stress the results in order to validate their ability to generalize. The classification is performed several times by using smaller subsets of the original data. Finally, the results are combined together to obtain the definitive classification performance. This procedure makes the model independent from the dataset and increases the probability that it will perform better with new data [20].

### 2.3. Data Preparation

Medical records were screened to identify the eligible patients. All numerical and categorical variables (independent variables) were used to create both linear and multiple regression models to study their relationship with UAS7 (response variable).

Concomitant diseases were organized into general groups to identify the most frequent associated diseases (Table 2).

The same approach was used for the abnormal blood test values (Table 3).

Outlier values from each variable were removed. After that, missing values were replaced with the mean value. Finally, the numerical variables were preprocessed and normalized with a scaling function.

Different ML models (e.g., k-NN, SVM, lasso, logistic, ridge and elastic net regression) that have been profitably applied for predicting clinical information were explored [18,19,20,21,22,23], to find a model that could predict the response to treatment with omalizumab in CSU patients. The final dataset was randomly subdivided into a training and a test set of two-thirds and one-third, respectively. The results were assessed with a 2-fold cross validation that was repeated 10 times.

The performance of each model was compared calculating the statistical measures e.g., accuracy, specificity, sensitivity, precision, F1 score [24].

The contribution of each variable was evaluated through Variable Importance Analysis (VIA) in R with both caret and Boruta packages. Model was trained with data scaling preprocessing, svmRadialWeights method and train control with repeatedcv resampling method.

### 2.4. Ethical Approval

The study was conducted according to the principles of the Declaration of Helsinki and approved informed consent was obtained from all patients.

## 3. Results

### 3.1. Concomitant Diseases

Results showed no correlation between concomitant diseases and the severity of urticaria (UAS7) or the response to post-treatment therapy.

It was observed that ER patients were predominantly characterized by hypertension, while LR and NR mainly suffered from asthma. Overall, rhinitis and dyslipidemia were common concomitant diseases. Conversely, hypothyroidism was found only in the group of NR patients. Between groups, not statistical difference was found in the number of concomitant diseases.

### 3.2. Treatment Efficacy

**Figure 1:**
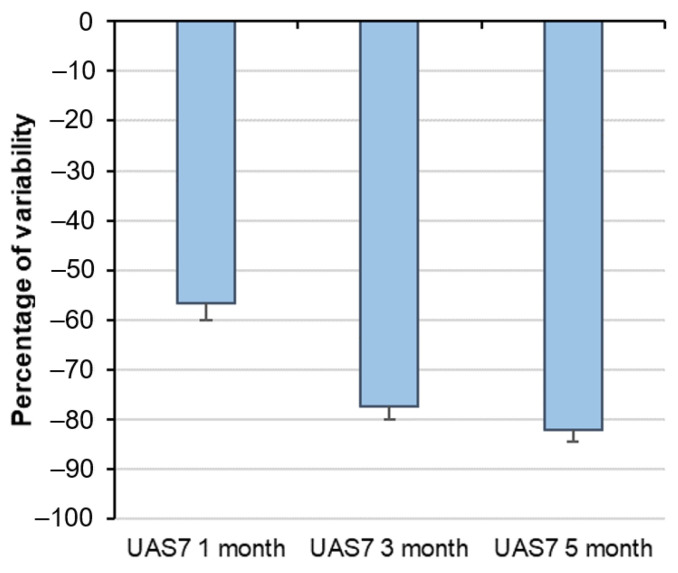
Variability of the response to Omalizumab over 5 months treatment.

Interesting, almost 60% of the CSU patients had a good response to omalizumab after 1 month treatment and, this percentage increased as far as more than 80% after 5 months treatment (Figure 1).

**Figure 2:**
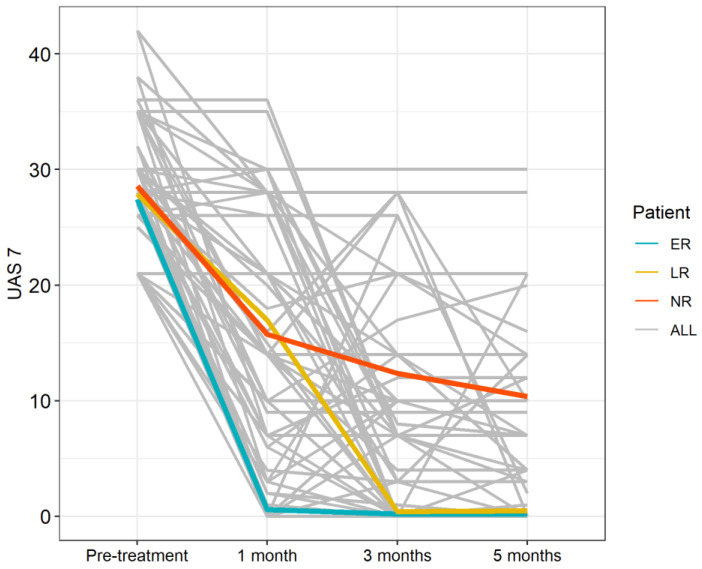
UAS7 Flow diagram according to CSU patient groups.

The clinical response of CSU patients before and after treatment with Omalizumab is shown in Figure 2.

**Figure 3:**
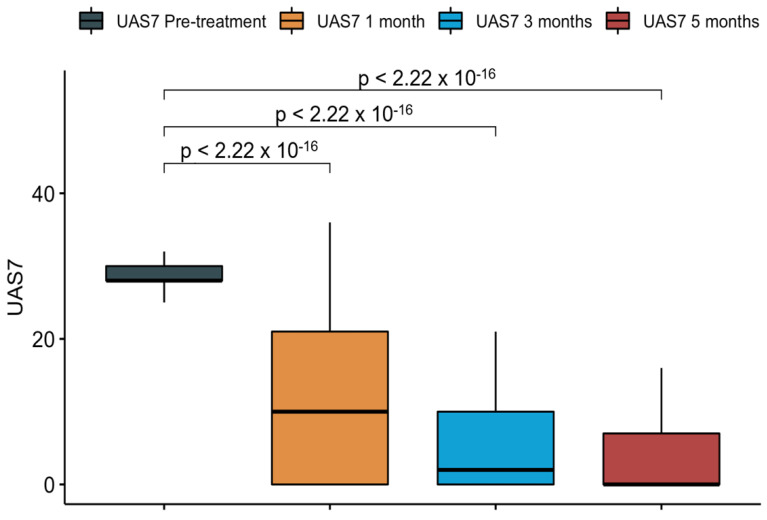
Response to omalizumab in South Italian CSU patients.

It was interesting to know, how the response to omalizumab has been over time. The scores obtained at 1, 3 and 5 months confirmed that the majority of patients responded moderately at the first month and almost completely, at third and fifth month (Figure 3) [25].

### 3.3. Response to Treatment Based on Patient’s Characteristics

It was also investigated whether gender, age, weight or height affected the severity of urticaria or the response to therapy. Only the variables height and age at 1 and 5 months, respectively, were found to be statistically significant for the response to treatment with omalizumab [26].

The place where patients live (inner city vs. countryside) affected neither the severity nor the duration of the disease.

### 3.4. Impact of Disease Duration

A positive, although limited correlation was found between the number of years the patient had been suffering from urticaria and the response to therapy. Conversely, CSU patients with angioedema were statistically related neither to the severity of the disease nor to the response to therapy.

### 3.5. Association between Total IgE Levels and Response to Omalizumab

It was found a slight positive correlation (R^2^ = 0.21) between total serum IgE levels and UAS7 at month 1, with no correlation at months 3 or 5.

## 4. Variable Importance Analysis

Based on patient data, the Variable Importance Analysis (VIA) assumes considerable significance for the selection of the best features and for the classification of performance improvement.

For this purpose, we considered personal patient information, disease duration, serological results and, finally, disease severity at 1, 3 and 5 months.

**Figure 4:**
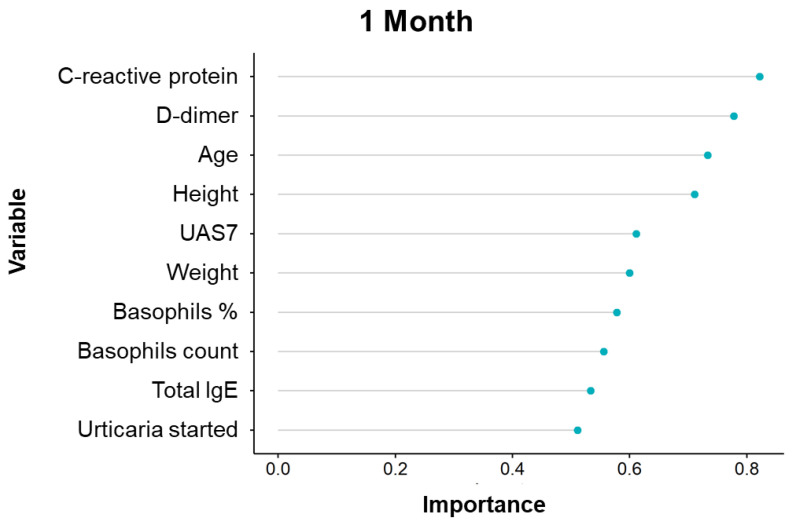
Variable Importance Analysis for the prediction of the response to omalizumab after 1 month treatment.

**Figure 5:**
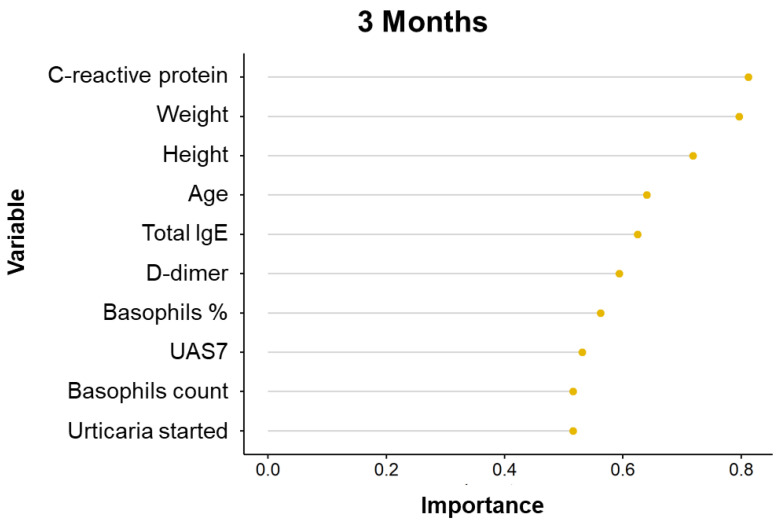
Variable Importance Analysis for the prediction of the response to omalizumab after 3 months treatment.

**Figure 6:**
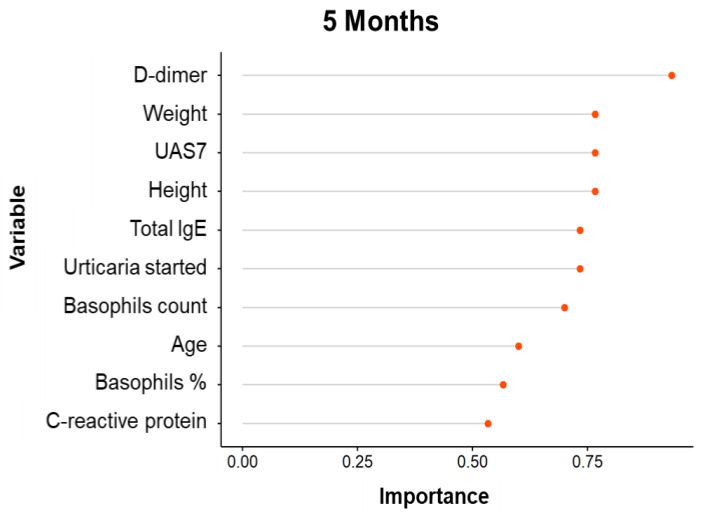
Variable Importance Analysis for the prediction of the response to omalizumab after 5 months treatment.

The importance of variables for the performance classification changes over time (Figure 4, Figure 5 and Figure 6).

However, many variables ranked similarly in the first three months of treatment, like D-dimer and *C*-reactive protein, followed by age, height, weight. By contrast, total serum IgE and basophils levels had a lower rank of importance.

**Figure 7:**
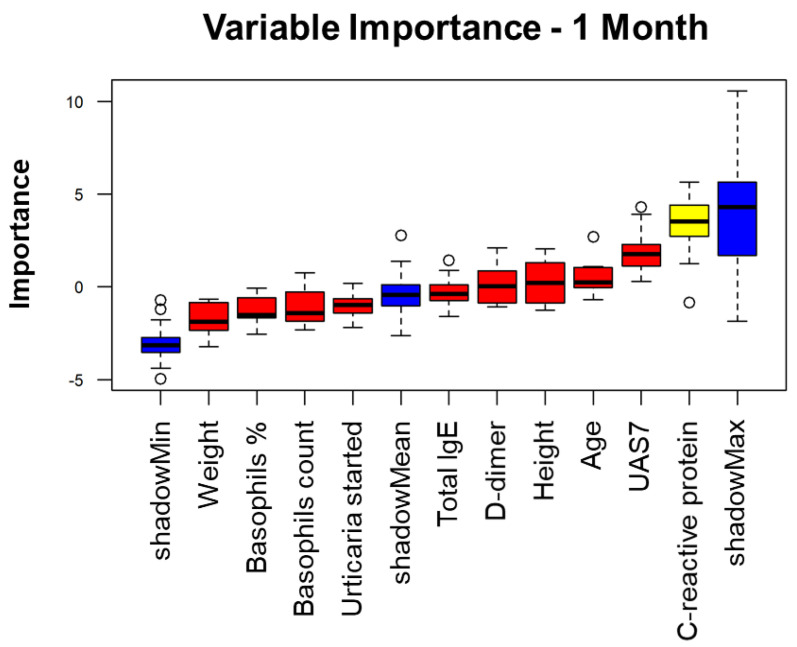
Feature selection algorithm based on importance.

Boruta method analysis showed that only *C*-reactive protein is significantly associated with treatment response at 1 month (Figure 7).

Basically, the variable analysis selects statistically and clinically relevant tests. It is important to consider these results for the follow up of CSU patients during omalizumab treatment.

## 5. Machine Learning Methods Classification

The overall results for each ML method are summarized in Table 4.

The SVM model was created by using onset of urticaria (years), total serum IgE Test, Basophils percentage and Basophils Counts, D-dimer, Reactive C-Protein and UAS7 pre-treatment. Several kernels were tested, e.g., linear, polynomial, radial basis and sigmoid. The best results were obtained with sigmoid kernel, reaching an increased performance. The cost of constraints violation was set to 100. Finally, for each temporal step, 1, 3 and 5 months of treatment, accuracy, sensitivity, specificity and precision measures were obtained.

Taken together, these results confirm the utility of the ML approach in learning from patient clinical records and suggest the use of feature selection through VIA as a powerful statistical tool.

## 6. Discussion

Electronic Medical Records (EMR) are a powerful source of information and temporal data that foster retrospective studies as the number of patients grows. The use of ML techniques in allergies is still being explored [27]. To our knowledge, this is the first time that a study explores the potential of ML approaches in predicting the response to omalizumab in patients with chronic spontaneous urticaria (CSU).

Moreover, these techniques help to identify the most important indicators through feature selection such as D-dimer and C-reactive protein.

Most patients treated with omalizumab respond quickly to treatment, although to varying degrees. The most selective classification methods (k-NN and SVM) are able to provide high accuracy but lower precision value. These results could be explained due to the intrinsic diversity of our cohort and, by extension, the original cause of urticaria and the way omalizumab affects each patient. Furthermore, there is a reduced number of patients with urticaria in the analyzed time interval because many of them responded early to omalizumab. As a consequence, many classifiers are able to identify very well the true negative responses at the beginning, while accuracy tends to decrease, although, conversely, the true response rate increases in the third month. In this scenario, SVM represents the most stable approach.

Classical ML approaches are suitable for non-large datasets and hyperparameter optimization is easier to control. These algorithms can shed light on disease-specific traits by analyzing the most statistically relevant characteristics emerging from the data.

The results confirmed a mild robustness to potential bias [28] by revealing literature-based characteristics of CSU patients. Feature selection starts from clinical practice being able to suggest new examinations and tests not previously linked to the disease, with the aim to predict the outcome or the response to treatment in novel patients. It is important that future studies extend the analysis with ML approaches, considering as much information as possible; especially for diseases with unknown etiology like CSU.

Interesting, ML approaches showed good accuracy already in the third month of treatment with omalizumab and selected concomitant disease, disease duration and serological exams (IgE levels, D-dimer and C-reactive protein among others) were crucial characteristics that achieved a better performance. Having classified the patients into ER or LR was the best choice compared with other alternatives.

## 7. Conclusions

Nowadays, omalizumab is the only approved third-line treatment for patients with antihistamine refractory CSU [4]. ML techniques could be effectively used to predict the response to omalizumab therapy, extending our understanding about how it works [29,30].

Further studies involving transcriptional levels could broaden this landscape for the selection of new clinical biomarkers.

## Figures and Tables

**Table 1:** Selected baseline variables from 132 South Italian CSU patients.

	ER	LR	NR
Female/Male (%)	57/43	65/35	67/33
Age (ys)	45.8	50.5	50.4
Disease duration (ys)	5.7	5.1	6.2

**Table 2:** Concomitant diseases in South Italian CSU patients.

Concomitant Diseases	Patients *n* = 132
Respiratory	37
Thyroid	24
Hypertension	23
Dislipidemia	13
Autoimmune	12
Gastrointestinal	12
Allergies	7
Psychiatric	9
Miscellaneous	16

**Table 3:** Most frequent abnormal blood test from South Italian CSU patients.

Blood Test	Total Patients (%)
Total IgE	18.9
Anti-TPO	12.9
CRP	10.6
D-dimer	9.8
ANA	6.1
Anti-Tg	6.1
Eosinophilia	5.3
ESR	3.8
ANCA	2.3
Glycemia	2.2
H. pylori	2.3
Specific IgE (cat)	1.5
Others	6

**Table 4:** Prediction of the response to treatment with omalizumab using 5 different ML methods.

Months	Accuracy	Sensitivity	Specificity	Precision	Method
1	0.631647	0.325238	0.801691	0.464167	Elastic net
3	0.483874	0.552738	0.473431	0.44404	Elastic net
5	0.493051	0.689466	0.279524	0.542756	Elastic net
1	0.71	0.171429	1	1	k-NN
3	0.473684	0.8	0.236364	0.433333	k-NN
5	0.5	1	0	0.5	k-NN
1	0.631647	0.325238	0.801691	0.464167	Lasso
3	0.483874	0.552738	0.473431	0.44404	Lasso
5	0.493051	0.689466	0.279524	0.542756	Lasso
1	0.362471	0.658095	0.198309	0.318586	Logistic
3	0.516126	0.447262	0.526569	0.419524	Logistic
5	0.506949	0.310534	0.720476	NA	Logistic
1	0.61672	0.26	0.818475	0.386191	Ridge
3	0.48104	0.563691	0.46434	0.439895	Ridge
5	0.495892	0.700577	0.267024	0.539624	Ridge
1	0.6022222	0.375	0.7533333	0.3541667	SVM
3	0.7666667	0.6875	0.8133333	0.5925926	SVM
5	0.315	0.5208333	0.3703704	0.4351852	SVM

## Data Availability

Not applicable.

## Abbreviations

CSU	Chronic spontaneous urticaria
UAS7	Urticaria Activity Score over 7 days
ML	Machine learning
ER	Early responder
LR	Late responder
NR	Non-responder
GERD	Gastroesophageal reflux disease
IgE	Immunoglobulin E
IBS	Irritable bowel syndrome
SNAS	Systemic nickel allergy syndrome
BPH	Benign prostatic hyperplasia
TPO	Thyroid peroxidase antibody
CRP	C-reactive protein
ANA	Anti-nuclear antibody
Tg	Thyroglobulin
ANCA	Anti-neutrophil cytoplasmic antibodies
ESR	Erythrocyte sedimentation rate
SVM	Support Vector Machine
k-NN	k-nearest neighbors

## References

[1] Fok J.S., Kolkhir P., Church M.K., Maurer M. (2021). Predictors of treatment response in chronic spontaneous urticaria. Allergy.

[2] O’donnell B.F., Lawlor F., Simpson J., Morgan M., Greaves M. (1997). The impact of chronic urticaria on the quality of life. Br. J. Dermatol..

[3] Staubach P., Eckhardt-Henn A., Dechene M., Vonend A., Metz M., Magerl M., Breuer P., Maurer M. (2006). Quality of life in patients with chronic urticaria is differentially impaired and determined by psychiatric comorbidity. Br. J. Dermatol..

[4] Zuberbier T., Aberer W., Asero R., Bindslev-Jensen C., Brzoza Z., Canonica G.W., Church M.K., Ensina L.F., Giménez-Arnau A., Godse K. (2018). The EAACI/GA^2^LEN/EDF/WAO guideline for the definition, classification, diagnosis and management of urticaria. Allergy.

[5] Maurer M., Altrichter S., Bieber T., Biedermann T., Braeutigam M., Seyfried S., Brehler R., Grabbe J., Hunzelmann N., Jakob T. (2011). Efficacy and safety of omalizumab in patients with chronic urticaria who exhibit IgE against thyroperoxidase. J. Allergy Clin. Immunol..

[6] Zhao Z.-T., Ji C.-M., Yu W.-J., Meng L., Hawro T., Wei J.F., Maurer M. (2016). Omalizumab for the treatment of chronic spontaneous urticaria: A meta-analysis of randomized clinical trials. J. Allergy Clin. Immunol..

[7] Maurer M., Rosén K., Hsieh H.-J., Saini S., Grattan C., Gimenéz-Arnau A., Agarwal S., Doyle R., Canvin J., Kaplan A. (2013). Omalizumab for the Treatment of Chronic Spontaneous or Spontaneous Urticaria. N. Engl. J. Med..

[8] Metz M., Ohanyan T., Church M.K., Maurer M. (2014). Omalizumab is an effective and rapidly acting therapy in difficult-to-treat chronic urticaria: A retrospective clinical analysis. J. Dermatol. Sci..

[9] Maurer M., Giménez-Arnau A.M., Sussman G., Metz M., Baker D.R., Bauer A., Bernstein J.A., Brehler R., Chu C.-Y., Hung S.-I. (2019). Ligelizumab for Chronic Spontaneous Urticaria. N. Engl. J. Med..

[10] Deo R.C. (2015). Machine Learning in Medicine. Circulation.

[11] Segura-Bedmar I., Colón-Ruiz C., Tejedor-Alonso M.A., Moro-Moro M. (2018). Predicting of anaphylaxis in big data EMR by exploring machine learning approaches. J. Biomed. Inform..

[12] Moreno-Indias I., Lahti L., Nedyalkova M., Elbere I., Roshchupkin G., Adilovic M., Aydemir O., Bakir-Gungor B., Pau E.C.-D.S., D’Elia D. (2021). Statistical and Machine Learning Techniques in Human Microbiome Studies: Contemporary Challenges and Solutions. Front. Microbiol..

[13] Connor C.W. (2019). Artificial Intelligence and Machine Learning in Anesthesiology. Anesthesiology.

[14] De Gregory K.W., Kuiper P., De Silvio T., Pleuss J.D., Miller R., Roginski J.W., Fisher C.B., Harness D., Viswanath S., Heymsfield S.B. (2018). A review of machine learning in obesity. Obes. Rev..

[15] Patel L., Shukla T., Huang X., Ussery D.W., Wang S. (2020). Machine Learning Methods in Drug Discovery. Molecules.

[16] Messinger A.I., Luo G., Deterding R.R. (2019). The doctor will see you now: How machine learning and artificial intelligence can extend our understanding and treatment of asthma. J. Allergy Clin. Immunol..

[17] Eckman J.A., Hamilton R.G., Gober L.M., Sterba P.M., Saini S.S. (2008). Basophil phenotypes in chronic spontaneous urticaria in relation to disease activity and autoantibodies. J. Investig. Dermatol..

[18] Chang C.C., Lin C.J. (2011). LIBSVM: A library for support vector machines. ACM Trans. Intell. Syst. Technol. (TIST).

[19] Cover T., Hart P. (1967). Nearest neighbor pattern classification. IEEE Trans. Inf. Theory.

[20] Lever J., Krzywinski M., Altman N. (2016). Points of significance: Model selection and overfitting. Nat. Methods.

[21] Huang C., Clayton E.A., Matyunina L.V., McDonald L.D., Benigno B.B., Vannberg F., McDonald J.F. (2018). Machine learning predicts individual cancer patient responses to therapeutic drugs with high accuracy. Sci. Rep..

[22] Huang E.W., Bhope A., Lim J., Sinha S., Emad A. (2020). Tissue-guided LASSO for prediction of clinical drug response using preclinical samples. PLoS Comput. Biol..

[23] Fukushima A., Sugimoto M., Hiwa S., Hiroyasu T. (2019). Elastic net-based prediction of IFN-β treatment response of patients with multiple sclerosis using time series microarray gene expression profiles. Sci. Rep..

[24] Luo W., Phung Q.-D., Tran T., Gupta S., Rana S., Karmakar C., Shilton A., Yearwood J.L., Dimitrova N., Ho T.B. (2016). Guidelines for developing and reporting machine learning predictive models in biomedical research: A multidisciplinary view. J. Med. Internet Res..

[25] Kaplan A., Ferrer M., Bernstein J.A., Antonova E., Trzaskoma B., Raimundo K., Rosén K., Omachi T.A., Khalil S., Zazzali J.L. (2015). Timing and duration of omalizumab response in patients with chronic spontaneous/spontaneous urticaria. J. Allergy Clin. Immunol..

[26] Bousquet J., Rabe K., Humbert M., Chung K.F., Berger W., Fox H., Ayre G., Chen H., Thomas K., Blogg M. (2007). Predicting and evaluating response to omalizumab in patients with severe allergic asthma. Respir. Med..

[27] Platts-Mills T.A.E., Perzanowski M. (2018). The use of machine learning to understand the relationship between IgE to specific allergens and asthma. PLoS Med..

[28] Anto J.M., Bousquet J., Akdis M., Auffray C., Keil T., Momas I., Postma D.S., Valenta R., Wickman M., Cambon-Thomsen A. (2017). Mechanisms of the Development of Allergy (MeDALL): Introducing novel concepts in allergy phenotypes. J. Allergy Clin. Immunol..

[29] Vidyasagar M. (2015). Identifying Predictive Features in Drug Response Using Machine Learning: Opportunities and Challenges. Annu. Rev. Pharmacol. Toxicol..

[30] Gianfrancesco M.A., Tamang S., Yazdany J., Schmajuk G. (2018). Potential Biases in Machine Learning Algorithms Using Electronic Health Record Data. JAMA Intern. Med..

